# Syntactic Priming in American Sign Language

**DOI:** 10.1371/journal.pone.0119611

**Published:** 2015-03-18

**Authors:** Matthew L. Hall, Victor S. Ferreira, Rachel I. Mayberry

**Affiliations:** 1 Linguistics, University of Connecticut, Storrs, Connecticut, United States of America; 2 Psychology, UC San Diego, San Diego, California, United States of America; 3 Linguistics, UC San Diego, San Diego, California, United States of America; University of Barcelona, SPAIN

## Abstract

Psycholinguistic studies of sign language processing provide valuable opportunities to assess whether language phenomena, which are primarily studied in spoken language, are fundamentally shaped by peripheral biology. For example, we know that when given a choice between two syntactically permissible ways to express the same proposition, speakers tend to choose structures that were recently used, a phenomenon known as *syntactic priming*. Here, we report two experiments testing syntactic priming of a noun phrase construction in American Sign Language (ASL). Experiment 1 shows that second language (L2) signers with normal hearing exhibit syntactic priming in ASL and that priming is stronger when the head noun is repeated between prime and target (the lexical boost effect). Experiment 2 shows that syntactic priming is equally strong among deaf native L1 signers, deaf late L1 learners, and hearing L2 signers. Experiment 2 also tested for, but did not find evidence of, phonological or semantic boosts to syntactic priming in ASL. These results show that despite the profound differences between spoken and signed languages in terms of how they are produced and perceived, the psychological representation of sentence structure (as assessed by syntactic priming) operates similarly in sign and speech.

## Introduction

Sign languages provide researchers unique opportunities for examining the nature of human language, both in terms of what language is (linguistic analysis of language structure) and how it works (psycholinguistic studies of language processing). Linguistic analysis has provided rich descriptions of sign language structure at the phonological, lexical, and syntactic levels. Likewise, psycholinguistic experiments have supplied processing evidence for sign language at the phonological and lexical levels; however, sentence-level evidence is lacking, especially in production. The present study addresses this gap by investigating syntactic priming during online sentence production in American Sign Language (ASL). First, we briefly review the linguistic and psycholinguistic evidence from which the present study proceeds.

Linguistic analysis of the sign language lexicon has provided detailed descriptions of the morpho-phonological structure of signs [1–4]. A core observation that holds across various analyses of (sub)lexical structure in sign languages is that whole signs can be decomposed into smaller units of handshape, movement, and location, which are themselves recombined in principled ways to form an unbounded inventory of lexical forms [5]. In this way, these sign parameters can be seen as formally analogous to phonological features like manner and place of articulation in the sublexical structure of spoken language.

Building on these linguistic observations, psycholinguistic studies of lexical access in sign language have allowed the observation of the processes by which signers draw on their mental representations of these parameters for real-time lexical retrieval in both comprehension and production. For example, experimental measures (e.g. primed lexical decision, gating studies, etc.) find that comprehension behavior changes when handshape, location, and movement are manipulated [6–11]; the same is true for phonological neighborhood density [6]. In production, phonological facilitation is also observed [12], and naturally-occurring sign errors and sign-finding difficulty also confirm that handshape, location, and movement guide lexical retrieval [13–16]. In sum, these studies find that the principles that govern (sub)lexical structure are highly similar in sign and speech, both structurally and functionally.

The same kinds of investigations can be aimed at the syntactic level. Here again we find rich descriptions of syntactic structure in sign languages (for accessible overviews, see [17–19]). These analyses find many syntactic devices that are familiar from spoken language research (e.g. hierarchical phrase structure, constituent order, movement operations, anaphora, embedding, etc.), but there are other syntactic devices that make use of the unique affordances of the manual modality to accomplish grammatical functions (e.g. facial marking for topics and interrogatives, spatial marking for verb agreement, etc.). Furthermore, there are phenomena that are widespread in sign languages but unattested in spoken languages, such as the primacy of object agreement over subject agreement [20–23], and it has even been suggested that some sign languages allow rightward movement [24–26], which has long been disallowed under prevailing syntactic theory [27]. Thus, while there are certainly many parallels between spoken and signed syntax, we cannot take for granted that they are identical.

Likewise, we cannot assume that the psycholinguistic processes involved in syntactic operations are the same in signers as in speakers. In contrast to the wealth of linguistic description of syntactic structure in sign languages, there are fewer studies on the mental representation of syntax in signers. The earliest evidence for the psychological reality of signed syntax comes from studies of signers with left hemisphere brain damage. These studies find that similar syntactic deficits arise in response to damage to the classical left perisylvian language areas in both signers and speakers [28–31]. Interestingly, in signers with left hemisphere damage, these syntactic deficits include disruptions to *grammatical* use of space, such as maintaining spatial loci to accomplish anaphoric reference. Meanwhile, *topographical* use of space (e.g. describing the layout of a room, or a route from A to B) is intact following left hemisphere damage, but impaired after right hemisphere damage [32].

Additional studies have investigated the neural underpinnings of the linguistic structure of sign languages with the majority finding broad similarities between signed and spoken language when the language is learned from birth [33–35] although there may be some differences for hearing signers [36–37]. However, because the focus of our present study is on the behavioral evidence for the processing of syntactic structures in sign language, a detailed exposition of neuroimaging work on the topic is outside the scope of the present paper.

Despite this large amount of previous research, we still lack studies of the psycholinguistic processes involved in *producing* a signed sentence in real time. The present study aims to address this gap by presenting the results of two experiments on syntactic priming during sentence production in American Sign Language (ASL), which we turn to next.

## Syntactic Priming

The psychological reality of syntactic representations cannot be taken for granted. Although such descriptive devices are mainstays of formal linguistic analysis, our senses do not directly perceive abstract, hierarchical syntactic structures (e.g. noun phrases, c-command domains, etc.), either in speech or in sign. Instead, we only hear (or see) lexical items unfolding in linear sequences; any syntactic structure of comprehended sentences, if represented at all, must be inferred. (In fact, as a reviewer of an early version of this manuscript pointed out, even lexical items are not heard/seen directly, but are rather abstracted from acoustic/visual input.) It is therefore necessary to demonstrate their psychological reality in processing.

One way to do so is to test for evidence of a hypothesized structure independently of its perceptible content. This insight, first proposed by Bock [38], led to the discovery of syntactic priming. She reasoned that if syntactic structure is psychologically real, then exposure to a particular structure might increase the likelihood of that structure being chosen again, even if none of the actual words overlap between the prime sentence and the target sentence. In order for this conjecture to be testable, the language under study must provide a grammatical alternation, where (roughly) the same proposition can be expressed by more than one syntactic structure. The experimenter then creates stimuli that will elicit one of these alternatives (e.g., the English active/passive alternation, “Lightning struck the church”, “The church was struck by lightning”), and then exposes the participants to either an active or passive prime sentence before each target. The crucial finding, now replicated many times over (for a review, see [39]) is that participants are more likely to use a passive sentence to describe the target if the prime was passive than if the prime was active. The size of this difference is termed the priming effect. The main goal of Experiment 1 is to test whether syntactic priming is attested for signers. If so, it would suggest that signers, like speakers, make use of abstract syntactic representations when formulating utterances. Although there is no *a priori* reason to expect that signers would differ from speakers in this regard, it has not yet been demonstrated. Furthermore, while syntactic priming of the particular alternation studied here (described below) has been studied also in many spoken languages (a design choice that we thought was judicious), establishing that syntactic priming occurs in this familiar context is a necessary first step that opens the way for investigating syntactic alternations in sign languages that are understudied or unattested in spoken languages (e.g. wh-doubling, subject copy, optional agreement).

**Fig 1:**
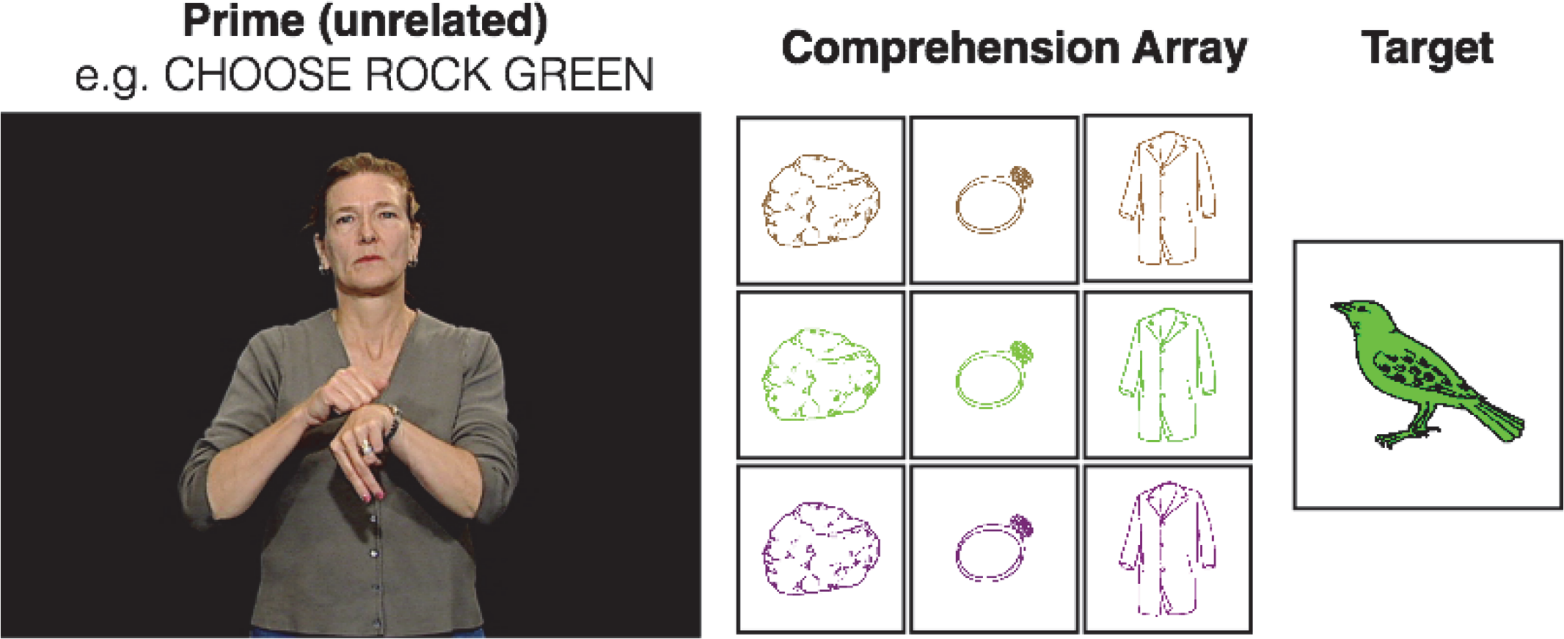
Example stimulus array during a syntactic priming trial. A video of the stimulus model signs the prime “CHOOSE ROCK GREEN.” The participant (not shown) chooses from an array of 3 different objects shown in 3 different colors. Next appears the target, which can be described as BIRD GREEN (post-nominal) or GREEN BIRD (pre-nominal). The individual in this figure has given written informed consent (as outlined in PLOS consent form) to publish these case details.

American Sign Language does not offer syntactic alternations for dative or active/passive structures, which are well studied in spoken languages. However, ASL does permit certain attributive adjectives to either precede or follow nouns [40]. MacLaughlin [40] analyzes pre-nominal adjectives (e.g., GREEN BIRD) as occupying the specifier position of the noun phrase, which is consistent with their receiving a modifier interpretation (“a/the green bird”). Post-nominal adjectives are analyzed as right-adjoined within a determiner phrase, which is consistent with the fact that they generally receive a predicative interpretation (“the bird that’s green”), although other analyses are possible. The details of this particular syntactic analysis are less important than the fact that the two structures are demonstrably different. Cleland and Pickering [41] showed that a similar alternation in English exhibits syntactic priming (“the red chair” vs. “the chair that’s red”), and Bernolet, Harsuiker, and Pickering [42] extended the paradigm to Dutch-English and German-English bilinguals. Therefore, Experiment 1 below is modeled after their studies. Briefly, participants watch a video of a native signer instructing them to choose a specific item (e.g. “CHOOSE CAR BLUE”) out of an array containing three blue objects and three cars in different colors (Fig. 1). Next, the participant saw a target shape (e.g. a red cow), and instructed the experimenter to choose that object out of a similar array by describing both its identity and its color. Priming is evidenced to the extent that post-nominal target descriptions are more common after post-nominal prime descriptions than after pre-nominal prime descriptions.

Since Bock’s seminal experiments, the syntactic priming paradigm has been used to probe a number of questions about sentence processing [39]. As these studies have accumulated, one effect that reliably emerges is a tendency for syntactic priming to be stronger when a phrasal head is repeated between the prime and target sentences. The reliability of this “lexical boost” has become established enough to be considered a signature effect of syntactic priming, although its mechanism is not fully understood. One account explains the lexical boost by suggesting that the use of a particular lexical item in a particular structure leaves traces in the system that make that structure more accessible the next time that same lexical item is retrieved [43]. Another account posits that the lexical boost is generated by episodic memory processes that fall outside of the core of syntax [44]. Our study is not designed to discriminate between these accounts; if the signers in Experiment 1 show a lexical boost, it could be due to either of these mechanisms.

Two further effects have been explored in previous research: a semantic boost and a phonological boost. The term “semantic boost” refers to an increase in the strength of the syntactic priming effect when the prime and target are semantically related. An example from noun phrase priming would be stronger priming to the target PIG after the prime COW than after the prime HAMMER. This effect was first observed by Cleland and Pickering (2003), who interpreted it as being consistent with the general consensus in models of sentence production that activation flows from conceptual levels to lexical levels, where syntactic information is integrated with lexical selection. To date, there is one published replication of the within-language semantic boost, also in English speakers [45].

The term “phonological boost” refers to an increase in the strength of the syntactic priming effect when the prime and target are phonologically related. An example in English would be stronger priming to the target “sheep” after the prime “sheet” than after the prime “ball”. This comparison was first made by Cleland and Pickering [41], who did not find evidence of any such boosts using minimal pairs like “sheep” and “sheet”. They concluded that activation at the phonological level did not influence the selection of syntactic structures, which is consistent with feed-forward models of language production [46]. However, a recent study by Santesteban, Pickering, and McLean [45] using homophones did find that syntactic priming increased if the prime and target head noun shared all of their phonology. They concluded that activation at the phonological level *can* influence the selection of syntactic structures, which is consistent with models of sentence production that allow feedback from phonological to lexical level processes, including syntactic selection [47]. However, they acknowledge that the data can also be explained by a comprehension-based account in which participants covertly activate *both* meanings of a homophone during initial processing stages, with activation in the production system flowing unidirectionally.

Experiment 1 tests whether syntactic priming occurs in ASL and whether there is a lexical boost. Experiment 2 tests for semantic and phonological boosts, and also manipulates age of ASL acquisition in the participants: as a first language in infancy (native L1 signers), as a first language later in childhood (non-native L1 signers), or as a second language in adulthood (hearing L2 signers).

## Experiment 1

As a first step, we recruited hearing participants who spoke English natively and had acquired a high degree of proficiency in ASL as a second language (L2). Several recent studies have shown that spoken-language bilinguals do show syntactic priming when tested entirely in their L2 [42, 48–50]. On the basis of these studies, it is reasonable to expect that syntactic priming should also occur in bilinguals whose L2 is signed, rather than spoken. However, to our knowledge, ours is the first study to test whether this is in fact the case. Furthermore, while priming within L2 has been consistently observed, the lexical boost has not always been present [50]. Therefore, the goal of Experiment 1 is to test for the existence of syntactic priming in a sign language, and whether there is a lexical boost in L2 signers.

### Method

All experimental procedures were approved by the institutional review board at UC San Diego. Study procedures were explained and informed consent was requested in either spoken English or ASL, according to the participants’ preference. Consenting participants then signed a form written in English.

**Participants.** We recruited 16 hearing non-native L2 signers (14 female; mean age = 31) who were proficient in ASL, in that they had been signing outside the classroom for at least 5 years. Ten of the sixteen were certified interpreters, and all 16 were involved with the Deaf community on a daily or weekly basis.

**Materials.** Following Cleland and Pickering [41], we selected line drawings of familiar objects and digitally manipulated their color, such that we could elicit either Noun-Adjective or Adjective-Noun descriptions from our participants. Most pictures were selected from the International Picture Naming Project (IPNP; [51]), with several additional pictures collected from the Internet or drawn by the first author in Microsoft PowerPoint for Mac 2004. (Pictures not available from the IPNP are available from the first author by request.) Because the IPNP does not contain norms for American Sign Language, the materials were selected by the first author and a team of native and non-native Deaf informants who aimed to choose items with the following characteristics: lexicalized (i.e. not fingerspelled, borrowed from Signed English, or a classifier), high name agreement (i.e. one dominant sign with little dialectal variation), judged likely to be known by non-native and hearing L2 signers, while retaining as many items as possible from Cleland and Pickering [41]. The list of prime-target pairs is provided as supplemental material.

For the colors, we selected blue, brown, green, purple, red, and yellow, because these colors are unambiguously adjectives in ASL (whereas the sign for the color orange and the fruit are the homophones).

Each target picture (e.g., a green bird) was matched with two types of prime nouns: unrelated (ROCK) or identical (BIRD). (By convention, glosses of ASL signs are written in capital letters.) The color of the prime and target nouns was always different. These prime nouns were then used in two types of sentence structures: pre-nominal (e.g. “CHOOSE GREEN ROCK”) or post-nominal (e.g. “CHOOSE ROCK GREEN”). Prime type (pre-nominal vs. post-nominal) and noun type (unrelated vs. identical) were factorially manipulated within subjects; which items appeared in which condition was counterbalanced across subjects. All stimuli were produced by a deaf native ASL signer. The signs we used are freely viewable on www.handspeak.com.

**Design and Procedure.** The testing session consisted of an exposure phase in which participants named the 96 experimental items in ASL, followed by a comprehension phase, and then the syntactic priming trials. Instructions were delivered via a pre-recorded movie of a deaf native ASL signer explaining each task. The first author, a fluent hearing signer, answered any questions in the participant’s preferred language.

In ASL picture naming, participants saw each of the 96 pictures, presented in black ink, and were instructed to name it using their preferred sign in its bare noun form. By administering this condition first, we aimed to obtain an unbiased measure of the participants’ preferred signs for the objects. This allowed us to correctly recognize their signs later in the syntactic priming trials if their sign was unexpected. If participants did not recognize what a picture was, the experimenter fingerspelled the object’s name and the participant then provided his or her lexical sign. If participants could recognize the picture but did not have a lexical sign for it, they typically fingerspelled its name. In the vast majority of cases, participants responded by producing a lexical sign, which was recorded on video. We did not take any quantitative measures of name agreement, but our materials elicited largely lexical signs with high name agreement. Note also that the syntactic priming effect may still be observed even if a participant does not use the dominant sign for an object. (For example, “The couch that’s red” is an acceptable post-nominal structure, even if the expected utterance was “The sofa that’s red.”)

Syntactic priming trials began with the videotaped stimulus model saying a brief ASL sentence of the form, “CHOOSE [x]”, where [x] could be either an Adjective-Noun phrase, a Noun-Adjective phrase, or the outline of a shape (on filler trials). This served as the prime sentence on critical trials. In all cases, the computer screen then displayed a 3 x 3 array of images. On critical trials, these were 3 objects (the correct noun and two foils), with each object appearing in 3 different colors. The array was systematic in that the objects were consistent by row and the colors were consistent by column; thus, starting with either the object name or the adjective was equally informative. After the participant clicked on a picture (using a mouse), the computer recorded their choice and then displayed a picture of a different colored object (the target) to the participant, who then described the target picture to the experimenter. During practice trials, the experimenter demonstrated to the participant that he too was looking at a 3 x 3 array of colored objects, so that he needed the participant to name both the object name and the color to choose successfully. Upon conclusion of the practice trials, the experimenter explained that he would no longer be showing the participant which object he was choosing, but participants were still under the impression that the task was a communicative one, when in reality the experimenter was recording whether the participant used a pre-nominal or post-nominal structure. This was done to incentivize the participants to continue to include both the noun and the color in their descriptions, as well as to mitigate any sense that their sentences were being judged by the experimenter. Participants’ responses were also recorded on video by a camera positioned next to the experimenter. (While signing, some participants directed their eyegaze to the camera, some to the experimenter, and others to the computer.)

There were 48 critical trials: each participant saw 24 pre-nominal primes and 24 post-nominal primes, which were each divided evenly between identical and unrelated nouns. The order of target pictures was constant for all participants, but the prime conditions were counterbalanced across participants in pseudo-random order, with the constraint that no one condition occurred more than 3 times in a row.

Intermixed with the 48 critical trials were 24 filler trials, where the participant’s task was similar, but no syntax was involved. Instead, the stimulus model described a shape, in accordance with ASL grammar, and the 3 x 3 array consisted of 9 shapes varying on 2 dimensions (e.g. size, thickness, rotation, reflection, etc.). These trials were much more difficult for participants, and were included to distract the participants from our critical trials. Data from these trials are not reported here.

### Results

We first excluded any trials that were neither pre-nominal nor post-nominal (0.5% of the data), and submitted the proportion of post-nominal descriptions to a 2 x 2 ANOVA with prime type (pre-nominal vs. post-nominal) and noun type (identical vs. unrelated) as within-subjects factors. These analyses were performed over subjects (F1) and items (F2). We also performed alternative analyses using linear mixed-effect models in R, using the maximal random effect structure that was justified by the data, as determined by model comparison. In this case, the inclusion of random effects was not justified, due to high correlations between fixed and random effects; thus, an intercept-only model was used. Both the raw data and our R code are available as supplemental material. Below, we report traditional ANOVAs over subjects (F1) and items (F2), results from linear mixed-effect models, and also use Monte Carlo simulations to confirm the reliability of specific effects (and non-effects) of interest. Monte Carlo (MC) simulations are a non-parametric approach that estimates probability by randomly reordering the observed responses across different conditions within participants and calculating how often the size of pseudo-effects in random simulations exceed the size of the observed effect. Repeating that process 1000 times yields a quantitative measure of probability: if randomly shuffled responses generate effects that are as large as or larger than the observed effects on more than 50/1000 simulations (*p* > .05), then the null hypothesis is not rejected. *P*-values from MC simulations are identified as such, and are given exact values to the thousandths place, which correspond to the number of simulations (out of 1000) where the shuffled data returned effects larger than those in the observed data.

**Fig 2:**
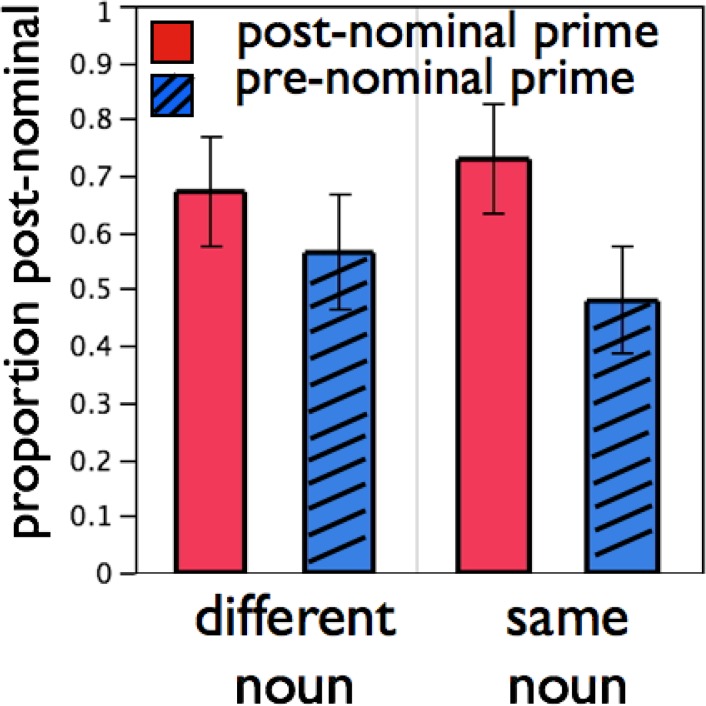
Syntactic priming with lexical boost in hearing L2 signers. Hearing L2 signers used post-nominal descriptions more after post-nominal primes (plain bars) than after pre-nominal primes (hashed bars), especially when the noun was repeated from prime to target. Error bars represent SEM.

As seen in Fig. 2, we found evidence of syntactic priming and of the lexical boost. When the prime and target nouns differed, signers produced 10.8% more post-nominal target descriptions after post-nominal prime descriptions than after pre-nominal prime descriptions. When the prime and target nouns were the same, this priming effect increased to 24.9%. The main effect of prime type [F1(1,15) = 15.00, *p* = 0.0 by MC simulation; F2(1,47) = 81.34, *p* = 0.0 by MC simulation] indicated that post-nominal descriptions were more common after post-nominal primes. There was also a prime type x noun type interaction [F1(1,15) = 8.29, *p* = .003 by MC simulation; F2(1,47) = 6.51, *p* = .004 by MC simulation], with stronger priming following identical nouns. This constitutes evidence for the lexical boost in ASL syntactic priming. A planned comparison of the effect of prime type within the unrelated condition was significant [t(15) = 3.07, *p* < .01], showing evidence of syntactic priming in the absence of lexical overlap in the noun phrase between prime and target. Finally, there was no main effect of noun type [F1(1,15) = 0.39, *p* = .54; F2(1,47) = 0.26, *p* = .61]. Linear mixed-effect analysis returned the same pattern of significant effects. Raw data and code are available in S1 Dataset.

### Discussion

When faced with a choice between two propositionally similar grammatical options, hearing L2 signers were significantly more likely to choose a structure that had been recently used, even without lexical overlap between prime and target. This is, to the best of our knowledge, the first demonstration of syntactic priming in a sign language and the first demonstration that signers, like speakers, draw on implicit syntactic representations during real-time sentence production. We also obtained evidence of a lexical boost: participants’ tendency to repeat the syntactic structure was exaggerated when the head noun was repeated between the prime and target. Due to some lexical variation in the signs produced by our participants, some prime-target pairs may have been closer to synonyms than to exact lexical repetitions. Synonyms have led to somewhat weaker activation in a study of English (as measured by ERPs; [52]), suggesting that the present data may, if anything, underestimate the size of the lexical boost effect.

However, it is worth remembering that the participants in Experiment 1 were all native English speakers who, though proficient in ASL, acquired it as a second language. It is possible that some or all of the syntactic priming effects observed in Experiment 1 were mediated through covert translation into English. Therefore, it is important to examine syntactic priming in deaf signers, including those who acquired ASL from birth (native L1 signers) as well as those who learned later in childhood (late L1 signers). A second group of hearing non-native L2 signers facilitates comparison to Experiment 1. Experiment 2 also affords an opportunity to test for two less-investigated aspects of syntactic priming: a phonological boost, and a semantic boost.

## Experiment 2

In addition to sign language being in a different modality than spoken language, the circumstances of language acquisition for congenitally deaf children are also unusual and create unique opportunities for researchers. Most deaf children are not exposed to sign language until several years after birth, ages by which most hearing children have already mastered their native language. Despite this situation being the norm, most psycholinguistic research on signers has focused on the native signing minority: the less than 10% of deaf children who are born into Deaf or signing families. There are good reasons for this; for example, it enables a more direct comparison between language acquisition for signers and speakers. Nevertheless, it is of both theoretical and practical interest to characterize the language processing systems of both native signers of ASL (the frequently-studied minority) and late L1 signers of ASL, the majority about whom less is known. (We acknowledge that ASL can be considered the native language of individuals who are born deaf and learn it after infancy outside the home. However, following established research convention, we refer to those individuals who learned ASL in infancy from their families as “native L1” signers and those who learned it after infancy outside the home as “late L1 signers”.)

Variation in the age onset of deaf learners’ sign language acquisition can be used to determine whether delayed L1 acquisition affects language proficiency in a fashion similar to or different from L2 learning of spoken languages. Delayed L1 effects have been found in deaf signer’s sentence processing across the various tasks of discourse shadowing [53], sentence memory [54–55], and grammatical judgment [56–58], as well as a variety of expressive tasks [59]. We note, however, that nearly all of these studies used off-line tasks, some of which require explicit metalinguistic judgments.

Syntactic priming may provide a way to tap into the kinds of implicit syntactic representations that underlie sentence production. The primary questions of Experiment 2 are whether deaf signers show evidence of syntactic priming, and whether the strength of the priming effect differs between native signers and non-native L1 signers. A group of hearing L2 signers is also included to facilitate comparison with Experiment 1.

In this experiment, we also explore two secondary questions concerning the mechanisms of syntactic priming in signers. As reviewed in the introduction, a number of studies of syntactic priming in speakers have manipulated the relationship between prime and target to gain insight into the mechanisms of syntactic processing in language production. Having found a lexical boost in Experiment 1, we now test for a phonological boost and a semantic boost.

The strongest phonological boost to syntactic priming was observed by Santesteban et al. [45], who used homophones as prime-target pairs. While the number of homophones in ASL is unknown and compilations of them are nonexistent, we can use sign-based minimal pairs to replicate Cleland and Pickering [41], who tested onset-related primes. There is no direct analogue of onset vs. rhyme relations in sign phonology, since the formational components of handshape, location, and movement overlap more in sign expression than do the phonemes of spoken words [62, 9–10]. When single parameters are shared between sign pairs in Spanish Sign Language, LSE, Carreiras et al. [6] found shared location to slow lexical decision relative to shared handshape. Also for LSE, Baus et al. [12] found shared location to slow picture naming. More akin to rhyming words in spoken language, however, are minimal pairs in sign language where two out of three parameters (handshape, location, and movement) are shared, which is the approach we take here. Shared movement and location has been found to speed lexical decision in British Sign Language, BSL, [7], and semantic category judgments of prime-target English words by deaf signers when the ASL translations are minimal pairs [60]. Effects of minimal sign pairs were not observed in “sign spotting” tasks done by deaf signers of BSL [11].

In addition, Santesteban’s [45] results suggested that maximizing the extent of overlap was a promising approach, and due to the fact that we needed both primes and targets to be picturable objects, we decided to maximize the number of shared features rather than attempt to control which features were shared. The result was that our phonologically related primes were minimal pairs of the target, differing in only one parameter, although the particular parameter varied unsystematically across items. We acknowledge that because nearly 80% of our phonologically related prime-target pairs shared location, this could potentially weaken the chances of detecting a phonological boost. We leave this issue for future research.

To test for a phonological boost to syntactic priming, Experiment 2 used half of the items from Experiment 1. For these 24 target items, we replaced the identical prime noun from Experiment 1 with a prime noun that was phonologically related in ASL. For example, the target BIRD was matched with the phonologically related prime NEWSPAPER, whose sign differs only in its location. The unrelated pairs were unchanged. Because many of the ASL signs denoting colors are themselves phonologically related (for example, GREEN, YELLOW, BLUE, and PURPLE differ only in handshape), we decided to control whatever contribution this relatedness might make by having all primes and targets share the same color in the related and unrelated conditions. Cleland and Pickering [41] found a small tendency for an increased priming effect when the adjective repeated between prime and target. More recently, an unpublished study by Scheepers [61] found a slight increase in syntactic priming when *any* content is repeated from prime to target. However, since the overlap in this case is the same in all conditions, it should not contaminate the questions of interest.

Experiment 2 used the remaining half of the items from Experiment 1 to test for a semantic boost to syntactic priming. For these 24 target items, we replaced the identical prime noun from Experiment 1 with a semantically related prime noun. If there is a semantic boost, signers should prime more to the related noun than to the unrelated noun, which remained unchanged from Experiment 1. For the sake of consistency, these items also used the same color adjective for both prime and target.

### Method

**Participants.** We tested 36 participants from three populations: deaf native L1 signers (n = 10; 7 female; mean age = 29), deaf late L1 signers (n = 10; 3 female; mean age = 36), and hearing non-native L2 signers (n = 16; 16 female; mean age = 29). Native L1 signers had at least one deaf and signing parent (n = 8), or an older deaf sibling in a hearing family where the parents chose to use ASL at home (n = 2). Late L1 signers were more heterogeneous in their background, but were crucially not immersed in a signing environment before age 3 (range 3–18, median = 8). Our intent in including this group was to examine the nature of syntactic representations in people with delayed exposure to a *first* language. Therefore, while it is difficult to retrospectively assess any adult’s early language development, we screened out prospective participants who reported being able to communicate with family or peers through spoken language before being exposed to sign, suggesting that they had more access to spoken language before learning to sign. In our initial response to potentially-qualified participants who identified themselves as late signers, we asked the following four questions:

1. What age were you first exposed to ASL?
2. What age were you first around other Deaf people who signed?
3. Before you learned sign, how did you communicate?
4. Is there anything else we should know about your language background?

Those who reported that they used speech and speechreading to communicate were screened out at this stage. However, several participants replied that despite being enrolled in oral education programs, they relied primarily on homesign-like systems for communication with family and friends before being exposed to sign. These participants were invited to participate, and filled out a more detailed questionnaire that asked more specific questions about their self-rated proficiency in production and comprehension of sign and speech at various stages of their lives and with various groups of interlocutors (family, friends, school, work, etc.). If these questionnaires had revealed substantial use of expressive and receptive spoken language prior to their reported age of exposure to ASL, we would have excluded these participants. However, none of our invited participants reported such ability. Thus, we cannot say with certainty that they had failed to acquire *any* fluency in English and may be better characterized as semi-lingual in English with some English fluency that does not surface in a speech or speechread form. Nonetheless, it is clear that none of the participants had full command of English as a primary language before being exposed to ASL. Aside from this screening, we did not independently manipulate late L1 versus late L2 among the non-native, deaf signers. The hearing L2 signers were all native speakers of English who had not been exposed to ASL during childhood (range 16–25, median = 19) but who had been signing ASL outside the classroom for at least 5 years. None had participated in Experiment 1.

All participants gave consent to participate and to be recorded on film, and were paid for their participation.

**Materials.** The targets and the unrelated primes were the same as those in Experiment 1. However, the primes from the identical condition of Experiment 1 were replaced with new primes, half of which were semantically related to the target (e.g. PIG-COW), with the other half phonologically related to the target (e.g. BIRD-NEWSPAPER). Phonological relatedness was operationalized as sharing two of the three major articulatory features in ASL (handshape, location, movement).

**Design and procedure.** As in Experiment 1, the testing session began with an exposure phase in which participants named all 96 prime and target images in ASL. Again, we found generally high name agreement across individuals and groups. The exposure phase continued with a sign comprehension task, in which each of the 96 signs was presented in isolation, followed by four pictures: the correct answer and three foils. Next was the syntactic priming task, followed by two other tasks that we do not discuss here: a phonological similarity judgment task (reported in Hall, Ferreira, & Mayberry, [62]) and a narrative comprehension task.

The design of the syntactic priming task was the same as in Experiment 1. All 48 target items were presented in a pseudo-random order, with the constraint that no one condition was repeated more than 3 times in a row. Items in the phonological set and semantic set were intermixed, with the 24 filler trials interspersed throughout the block.

### Results and Discussion

After excluding responses that were neither pre-nominal nor post-nominal (1% of the data), we carried out ANOVAs on the proportion of post-nominal descriptions produced, over both subjects (F1) and items (F2). In the subjects analysis, prime type (pre-nominal vs. post-nominal), stimulus class (phonological vs. semantic), and relatedness (related vs. unrelated) were within-subjects factors. Group (native, child L2, hearing L2) was a between-subjects factor. In the items analysis, group, prime type, and relatedness were within-items factors and stimulus class was a between-items factor. For linear mixed-effects analysis, an intercept-only model was the only one to successfully converge.

**Fig 3:**
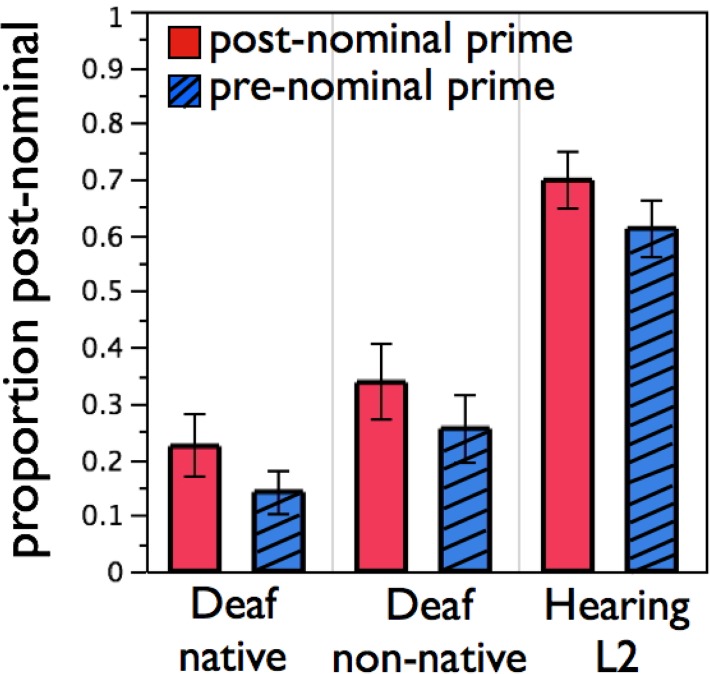
Equivalent syntactic priming across groups. Signers in each population produce more post-nominal descriptions after post-nominal primes (plain bars) than after pre-nominal primes (hashed bars). Error bars represent SEM.

Effects of interest are shown in Fig. 3. Evidence for syntactic priming comes from the main effect of prime type [F1(1, 33) = 7.17, p = 0.0 by MC simulation; F2(1,96) = 17.19, p = 0.0 by MC simulation]; participants were 8.4% more likely to produce a post-nominal description after a post-nominal prime (46.6%) than after a pre-nominal prime (38.2%). This effect is on the same general magnitude as in the unrelated condition in Experiment 1 (10.8%). Evidence that the groups were not differentially primed comes from the lack of any prime type x group interaction [F1(2,33) = 0.00, p = .69 by MC simulation; F2(2,94) = 0.04, p = .81 by MC simulation].

There was an unpredicted main effect of Group [F1(2,33) = 5.81, p < .01; F2(2,96) = 428.69, *p* < .001], indicating that some Groups used post-nominal structures more than others, regardless of Prime Type. In a post-hoc Tukey-HSD test, the only difference that was significant by both subjects and items was native L1 versus hearing L2 signers. Hearing L2 signers produced more post-nominal descriptions than did the native signers, despite the fact that its spoken analog (e.g. “The pig that’s red”) is dispreferred in English. Both of these main effects, and no others, were robustly significant in linear mixed-effect analysis.

The secondary questions of Experiment 2 concerned whether we would find a phonological and/or semantic boost to syntactic priming. We found neither. This is partially confirmed by the lack of a prime type x relatedness interaction [F1(1,33) = .17, *p* = .68; F2(2,96) = .01, *p* = .91], indicating that when collapsing across phonological and semantic items, related nouns did not yield more priming than unrelated nouns. The lack of boosts is further verified by the lack of a prime type x stimulus class x relatedness interaction [F1(1,33) = 1.94, p = .17; F2(1,96) = .84, p = .36], suggesting that phonological and semantic pairs were equally (un)affected by relatedness. These effects were also absent in linear mixed-effects analysis. We address these non-effects in the general discussion.

To rule out the possibility that the phonological boost was absent because we chose stimulus items without strong phonological relationships, or that our participants had different signs for target items than we intended (potentially disrupting the phonological relationship), we devised a phonological similarity judgment task, which was administered immediately following the syntactic priming task. It is described in detail in Hall, Ferreira, and Mayberry [62]. On 94.5% of trials, participants indicated that the related prime was in fact more phonologically similar to their own sign for the target than the unrelated prime. This suggests that the phonological similarity manipulation was robust.

The only other effect that approached significance in these analyses was unpredicted and difficult to interpret meaningfully. This was a 3-way interaction of prime type x group x stimulus class, which was significant by subjects [F1(2,33) = 4.05, p < .03] but not by items [F2(2,96) = 1.95, p = .15]. In linear mixed-effect analysis, it was marginally significant (z = 1.75, p = .08). Recall that stimulus class corresponds to the two different sets of materials designed to assess the semantic and phonological boosts respectively. This interaction arose because the native L1 signers showed stronger syntactic priming on the semantic items while the late L1 signers showed stronger syntactic priming on the phonological items. Note that the factor relatedness is not involved in this interaction; thus, whatever caused it did not vary as a function of whether the prime and target were semantically or phonologically related versus unrelated. Because these item sets did not differ in any *a priori* way, and due to the marginal nature of the effect, we refrain from attempting to interpret a potentially spurious result.

Table 1 provides the size and standard effort of the priming effect for each condition. The priming effect is the difference in the prevalence of postnominal target descriptions as a function of prime type. Positive values indicate more postnominal productions after postnominal primes. Raw data and analysis code are available in S2 Dataset.

**Table 1:** Priming effect by population and stimulus type.

Population	Stimulus Class	Relatedness	Priming Effect:Mean % (SEM)
Native	Phonological	Related	5% (2.6)
Native	Phonological	Unrelated	2% (1)
Native	Semantic	Related	10% (3.5)
Native	Semantic	Unrelated	16% (2.5)
Non-native L1	Phonological	Related	18% (.9)
Non-native L1	Phonological	Unrelated	8% (.9)
Non-native L1	Semantic	Related	0% (2)
Non-native L1	Semantic	Unrelated	6% (2.5)
Hearing L2	Phonological	Related	8% (.4)
Hearing L2	Phonological	Unrelated	12% (.9)
Hearing L2	Semantic	Related	6% (.7)
Hearing L2	Semantic	Unrelated	8% (.4)

## General Discussion

We have reported what we believe to be the first experiments demonstrating syntactic priming in a sign language. In Experiment 1, we showed that hearing L2 signers were more likely to use a post-nominal structure when they had recently seen a post-nominal prime, even if there was no lexical overlap between prime and target. We also showed that this effect increased if the head noun was repeated between prime and target, a phenomenon known as the lexical boost. In Experiment 2, we extended these results to deaf signers, including those who acquired ASL from infancy and those who learned ASL later in childhood. Despite their varied language backgrounds, the native L1, late L1, and hearing L2 signers did not differ in the strength of their syntactic priming effects. Finally, we found no evidence for a phonological or semantic boost to syntactic priming.

These results represent a first step toward characterizing online syntactic processing in signers. We find that, as in speakers, signers mentally represent more than a simple sequence of lexical items; they maintain implicit representations of grammatical structure. When a given structure has been recently encountered, the mental representation of that structure becomes more accessible for upcoming production than alternative structures. This new evidence extends previous findings by demonstrating that the parallels between sign language and spoken language are not restricted to the presence of linguistic structure, but extend to the psychological processing of syntactic structure as well.

The present study also sheds new light on how syntactic processing is (or isn’t) perturbed by non-native language acquisition. Previous studies have found that spoken-language bilinguals draw on their implicit representations of grammatical structures in L2 when producing sentences in real time [42, 48–50]. Experiment 1 demonstrated that these results extend to bilinguals whose second language is in a different modality (sign language). Experiment 1 also accords with the majority of these studies in finding a lexical boost even when priming entirely within L2.

An additional question that remains unresolved is whether there is a relationship between language proficiency and the amount of syntactic priming. Prior to our study, there were three contrasting findings. An unpublished study by Flett [49] compared the strength of syntactic priming of an active/passive alternation in Spanish with participants of varying proficiency: native speakers, intermediate L2 learners, and advanced L2 learners. She found significant priming in all populations; however, there was no monotonic relationship between priming and proficiency. Advanced L2 learners showed the most priming, followed by intermediate L2 learners, with native speakers showing the least. An additional complication to this study is the fact that intermediate L2 learners sometimes produced passive forms that would not be considered acceptable by a native speaker, which makes it difficult to interpret priming as reflecting the presence of well-formed syntactic representations.

A different approach to assessing the relationship between priming and proficiency was taken by Bernolet, Hartsuiker, and Pickering [48], who measured individual differences in the priming of an English genitive structure. Although there was a clear positive relationship between proficiency and priming from L1 to L2, the relationship was much less clear for priming entirely within L2. Bernolet et al. [48] found a weak positive relationship when the prime and target word were semantically unrelated; however, there was a significant negative relationship between proficiency and priming when the prime and target word were identical. The present finding of equal priming between our three groups of signers falls between these two previous results. With only three studies, each testing different alternations in different languages, more research is clearly needed to clarify the relationship between proficiency and syntactic priming.

The above studies, like most other work on syntactic priming in bilinguals, focus on native learners and hearing L2 learners, thus confounding proficiency with age of acquisition. No previous research has asked whether the ability to maintain syntactic representations is compromised by delayed acquisition of a *first* language. The results of Experiment 2 show that syntactic priming in late L1 signers was no stronger or weaker than in native L1 signers or hearing L2 learners, who also did not differ from one another. If a process as basic as syntactic priming had been perturbed by early language deprivation, the downstream consequences of that perturbation might have been able to account for other kinds of language difficulties that are commonly observed in non-native signers [56–57, 63].

We also asked whether the strength of the priming effect could be modulated by manipulating the relationship between the prime noun and target noun. As expected, priming was stronger if the prime and target nouns were identical (Experiment 1, the lexical boost). Priming was *not* stronger if the prime and target noun were phonological minimal pairs. While a phonological boost has been obtained with total phonological overlap [45], previous research with minimal pairs has not obtained a phonological boost to syntactic priming [41]. Although Bernolet, Hartsuiker, and Pickering [64] found that translation equivalent primes led to stronger priming if they were cognates (i.e. both semantically and phonologically related), it is not clear whether this result predicts a within-language phonological boost, or whether this only happens when the items are semantically related to begin with (whether within or between language). We leave this issue for future research.

More surprising was the absence of a semantic boost to syntactic priming. Both Cleland and Pickering [41] and Santesteban et al. [45] reported a small but significant increase in the syntactic priming effect when the prime and target nouns belonged to the same semantic category. In contrast, we found no such evidence, despite using very similar materials to those in Cleland and Pickering ([41]; see S1 Appendix). In the absence of any motivated reason to suspect modality differences to influence the semantic boost, we look to further research to clarify these questions, including replications in spoken languages other than English, and replications in sign language using more subjects and stimuli.

## Conclusions

There is little if any extant research measuring the implicit nature of syntactic representations during online sentence production in sign language. Here, we present some of the first evidence by testing whether signers, like speakers, are more likely to describe a picture using a given syntactic structure when that same structure was recently presented in a prime sentence. We found that signers did exhibit syntactic priming, and that the strength of priming did not vary as a function of hearing status or age of first language exposure. This, in turn, suggests that whatever factors underlie the grammatical difficulties observed in non-native signers, especially late L1 signers, they do not lie principally with an inability to extract and maintain psychological representations of simple syntactic structures.

## Supporting Information

S1 Appendix Prime-target pairs for Experiments 1 and 2. (DOCX)

S1 Dataset Raw data and R code for additional analysis of Experiment 1. (ZIP)

S2 Dataset Raw data and R code for additional analysis of Experiment 2. (ZIP)
