# Supplementary material for: Syntactic Priming in American Sign Language
Source: PLoS One. 2015 Mar 18;10(3):e0119611. doi: 10.1371/journal.pone.0119611 (PMC4364966; doi:10.1371/journal.pone.0119611)
Supplement: S1 Appendix — (DOCX) [file pone.0119611.s001.docx]

Appendix. Prime-Target pairs for Experiments 1 & 2.

| Target  (Expt 1 & 2) | Identical Prime (Expt 1) | Unrelated prime  (Expt 1 & 2) | Related Prime (Expt 2) | Stimulus class (Expt 2) |
| --- | --- | --- | --- | --- |
| BIRD | BIRD | ROCK | NEWSPAPER | Phonological (location) |
| BOOK | BOOK | BUTTER | BOAT | Phonological (movement) |
| CHAIR | CHAIR | BICYCLE | TRAIN | Phonological (movement) |
| CHEESE | CHEESE | SOAP | PAPER | Phonological (movement) |
| CHURCH | CHURCH | WINDOW | CHOCOLATE | Phonological (movement) |
| COMB | COMB | MOON | RAKE | Phonological (location) |
| DOCTOR | DOCTOR | DOLL | SOAP | Phonological (movement) |
| EAGLE | EAGLE | TYPEWRITER | DOLL | Phonological (orientation) |
| EGG | EGG | FLAG | KNIFE | Phonological (movement) |
| FISH | FISH | KNIFE | FLAG | Phonological (movement) |
| GLASSES | GLASSES | RAKE | MOON | Phonological (movement) |
| KEY | KEY | SCREWDRIVER | ONION | Phonological (location) |
| LAWNMOWER | LAWNMOWER | ONION | MOTORCYCLE | Phonological (movement) |
| NOSE | NOSE | STAR | MOUSE | Phonological (movement) |
| NURSE | NURSE | BOAT | BUTTER | Phonological (movement) |
| OWL | OWL | SHOWER | BINOCULARS | Phonological (handshape) |
| PIANO | PIANO | MOUSE | TYPEWRITER | Phonological (movement) |
| POTATO | POTATO | NEWSPAPER | ROCK | Phonological (handshape) |
| RAINBOW | RAINBOW | MOTORCYCLE | FENCE | Phonological (movement) |
| SCISSORS | SCISSORS | FENCE | LOBSTER | Phonological (# of hands) |
| SHOE | SHOE | TRAIN | BICYCLE | Phonological (movement) |
| SOCK | SOCK | BINOCULARS | STAR | Phonological (orientation) |
| SUN | SUN | DOLL | SHOWER | Phonological (movement) |
| UMBRELLA | UMBRELLA | PAPER | WINDOW | Phonological (handshape) |
| AIRPLANE | AIRPLANE | BASEBALL BAT | HELICOPTER | Semantic |
| BEAR | BEAR | LETTUCE | WOLF | Semantic |
| BROOM | BROOM | GHOST | VACUUM | Semantic |
| BUTTERFLY | BUTTERFLY | SNAKE | BUG | Semantic |
| CAKE | CAKE | MATCH | PIE | Semantic |
| CANDLE | CANDLE | PIE | MATCH | Semantic |
| COFFEE | COFFEE | BUG | TEA | Semantic |
| COW | COW | BOY | PIG | Semantic |
| FLUTE | FLUTE | HOT DOG | VIOLIN | Semantic |
| FOOTBALL | FOOTBALL | FORK | BASEBALL BAT | Semantic |
| GIRAFFE | GIRAFFE | VACUUM | ELEPHANT | Semantic |
| GIRL | GIRL | PIG | BOY | Semantic |
| HAMBURGER | HAMBURGER | VIOLIN | HOT DOG | Semantic |
| HAMMER | HAMMER | COAT | SCREWDRIVER | Semantic |
| HAT | HAT | SCREWDRIVER | COAT | Semantic |
| PRIEST | PRIEST | TEA | NUN | Semantic |
| RING | RING | FROG | NECKLACE | Semantic |
| SPIDER | SPIDER | NUN | SNAKE | Semantic |
| SPOON | SPOON | ELEPHANT | FORK | Semantic |
| TOAST | TOAST | FLOWER | BREAD | Semantic |
| TOMATO | TOMATO | WOLF | LETTUCE | Semantic |
| TREE | TREE | BREAD | FLOWER | Semantic |
| TURTLE | TURTLE | NECKLACE | FROG | Semantic |
| WITCH | WITCH | HELICOPTER | GHOST | Semantic |
